# Dual field magnetic separation for improved size fractionation of magnetic nanoparticles

**DOI:** 10.1039/d5nr02659h

**Published:** 2025-10-08

**Authors:** Manuel Wolfschwenger, Jonathan Leliaert, Aaron Jaufenthaler, Daniel Baumgarten

**Affiliations:** a Institute of Electrical and Biomedical Engineering, UMIT TIROL – Private University for Health Sciences and Health Technology 6060 Hall in Tirol Austria; b Department of Solid State Sciences, Ghent University 9000 Ghent Belgium; c Biomedical Engineering Group, Department of Mechatronics, University of Innsbruck 6020 Innsbruck Austria

## Abstract

Magnetic nanoparticles (MNPs) are emerging as key tools in biomedical and technical applications due to their tunable magnetic properties and responsiveness to external magnetic fields. However, the effectiveness of MNPs in applications such as targeted drug delivery, magnetic imaging and magnetic hyperthermia critically depends on achieving a narrow particle size distribution. Conventional gradient magnetic separation techniques often fall short in delivering high resolution size separation, particularly in the challenging 20 to 200 nm range, where the interplay between Brownian motion and magnetophoretic forces reduces separation precision. Therefore, in this study, we propose an enhanced gradient magnetic separation (GMS) method that superimposes a homogeneous alternating magnetic field onto an inhomogeneous gradient field and makes use of size-dependent magnetization dynamics. The proposed dual-field method is first verified in a simple test case, confirming that the desired separation behavior can principally be achieved. Simulations show that the magnetization ratio between particles of different sizes can be significantly increased beyond the predictions of the Langevin function. By systematically varying offset and alternating field strengths, an optimal combination maximizing this ratio is identified. Additionally, the influence of the alternating field frequency is investigated, showing that separation efficiency improves with increasing frequency up to a saturation point. To translate this behavior into effective spatial separation, particle trajectories are simulated while dynamically optimizing the alternating field strength over time to maximize the travelled distance ratio between large and small particles. The results demonstrate that large particles maintain strong alignment with the field, while smaller particles experience reduced time averaged magnetization, resulting in notably reduced mobility. Additionally, travelled distance ratios between particle sizes increase significantly compared to using a gradient field alone. The introduced dual-field method is also shown to remain effective for various particle sizes and under more realistic conditions where hydrodynamic and magnetic radii differ due to surface coatings. Finally, it is shown that the separation cut-off radius can be chosen arbitrarily, confirming the size independence of the method. These findings demonstrate that the proposed method substantially enhances size based separation, enabling improved control over particle size distributions and potentially advancing biomedical applications.

## Abstract 2

A dual-field magnetic separation method that significantly enhances size fractionation of magnetic nanoparticles, compared to conventional gradient-based methods.

## Graphical abstract

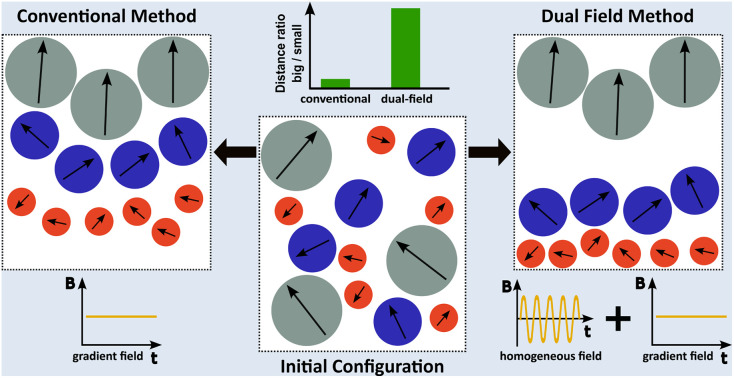

## Introduction

Magnetic nanoparticles have gained significant attention due to their unique properties and potential applications across various fields like biomedicine^1^ and technical disciplines.^2^ In medical applications, MNPs are widely used for targeted drug delivery,^3^ gene therapy,^4^ magnetic hyperthermia^5^ and imaging techniques such as magnetorelaxometry imaging (MRXI)^6,7^ and magnetic particle imaging (MPI).^8^ Their ability to respond to external magnetic fields enables precise localization and manipulation, making them ideal for minimally invasive treatments and diagnostics.^9^ On the technical side, MNPs are increasingly used in sensing,^10^ magnetic bearings,^11^ waste water treatment^12^ and separation processes.^13^

One of the most critical factors influencing the application performance is the particle size distribution.^14–16^ The physical and chemical properties of MNPs, such as magnetization, colloidal stability and biocompatibility, are highly dependent on the particle size.^1,9^ A broad size distribution can lead to undesirable aggregation,^17,18^ potentially hindering their function in biomedical applications and causing medical complications such as blood clotting and vessel blockage.^19^ Additionally, in imaging applications like MPI, a narrow size distribution is necessary to optimize spatial resolution and sensitivity.^15^

To obtain MNPs of specific sizes, various separation techniques have been developed. These include micro- and ultrafiltration,^20^ acoustic fractionation^21^ and centrifugation.^16^ Notably, Dadfar *et al*.^16^ successfully utilized centrifugation, achieving a significant improvement in MPI performance. Methods making use of magnetic field gradients are magnetic field-flow fractionation (mFFF), where nanoparticles, macromolecules and larger particles are separated by applying a field across a thin fluid channel^22,23^ and gradient magnetic separation.^24^ Gradient magnetic separation techniques, including high-gradient magnetic separation (HGMS) (>100 T m^−1^) and low-gradient magnetic separation (LGMS) (<100 T m^−1^), have emerged as promising strategies for separating MNPs based on their size and magnetic properties. Whereas HGMS has been used in various processes over the years,^25–28^ LGMS is the preferred strategy especially for biomedical applications due to the simplicity of the setup. Arsalani *et al*.^29^ successfully utilized LGMS to enhance the performance of MNPs as MPI tracers, confirming it as an efficient, reproducible and rapid method for MNP size selection. However, although the separation of MNPs from non-magnetic material has been widely explored, the aspect of size-based fractionation has received relatively less consideration in comparison, especially in mFFF.^30^

Despite significant advancements in MNP fractionation, challenges remain in achieving high-resolution size separation, particularly in the critical range of 20 to 200 nm. The overlapping influences of Brownian motion, convection and magnetic forces in this size range complicate the separation process, necessitating further research into novel fractionation strategies.^2^

In this work, we present a novel dual field magnetic separation method. Our goal is to enhance the conventional GMS method, thereby improving the separation efficiency of MNPs of varying sizes. In the study of Coene *et al*.^31^ it was demonstrated that particles of different sizes can be driven into two different regimes under the influence of an alternating magnetic field. Depending on the field strength, the particle magnetization was orientated either normal or parallel to the easy axis. This effect was then used to evaluate particle size distributions. Inspired by this concept, we extend this approach and use it for GMS. To explore its potential, we perform simulations of non-interacting particle ensembles subjected to homogeneous alternating magnetic fields, superimposed with inhomogeneous offset fields. As a result, large particles exhibit time averaged magnetizations that align almost entirely with the direction of the gradient field, while smaller particles show negligible magnetization components in that direction. Consequently, due to the magnetophoretic force acting in inhomogeneous fields,^32^ larger particles move along the gradient field, whereas smaller ones remain nearly stationary. This leads to an increased separation distance between particle types compared to conventional GMS, thereby enhancing the overall separation efficiency.

This paper is organized as follows: first, we introduce the basic concept and explain how our method is expected to improve conventional GMS. Next, the simulation model is presented, including the underlying physics and mathematical framework. To bridge the gap to practical application, we propose a corresponding simulation setup. This is followed by initial simulation results that demonstrate the principal feasibility of our method. Based on the setup, we then describe the procedure for evaluating the travelled distances of MNPs and conclude with examples involving particles both with and without a shell.

## Methodology

This section presents the theoretical framework of the magnetophoretic motion of MNPs, focusing on how magnetization and particle size affect their velocity in an external field. Building on this, we introduce a concept to enhance separation efficiency by driving particles of different sizes into distinct magnetization regimes.

### Magnetophoretic force and magnetization ratio

MNPs exposed to an inhomogeneous magnetic field experience a magnetophoretic force. The force expression depends on whether the electric current model or the magnetic charge model is used. In the case where 
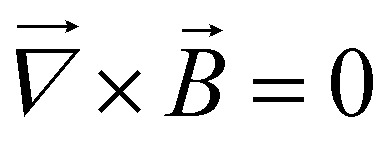
 both models agree and the force is given by^32^1
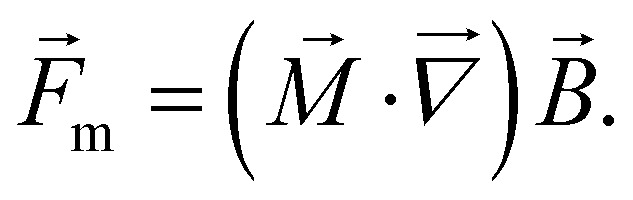


For single domain particles in a magnetic field, with a magnetic moment vector 
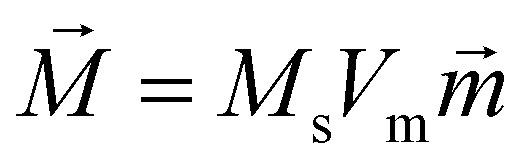
, this equation can be written as2
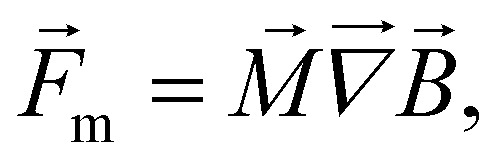
where 
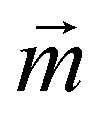
 is the direction vector of the particle's magnetic moment, *M*_s_ is the saturation magnetization and *V*_m_ is the particle's magnetic volume.

To describe the motion of a particle, it is important to consider the relevant fluid dynamics regime. According to Stoke's law,^33^ inertia effects can be neglected for Reynolds numbers Re < 1. For example, the Reynolds number of particles with *r*_h_ = 50 nm, which is common in ferrofluids,^34^ with a velocity *v* of 1 cm h^−1^ suspended in water at a temperature *T* of 300 K, is Re ≈ 3 × 10^−7^. Thus, the translational motion of a single particle in an inhomogeneous magnetic field can be described by the overdamped Brownian equation:3
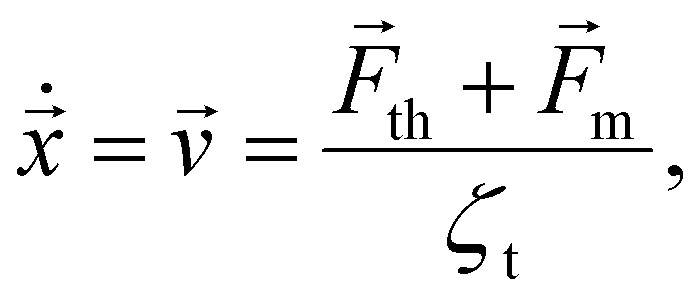
where 
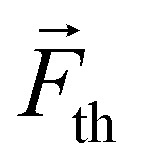
 is the thermal force, *ζ*_t_ = 6π*r*_h_*μ*_f_ ^33^ is the translational friction coefficient, *r*_h_ is the hydrodynamic particle radius and *μ*_f_ is the solvent's dynamic viscosity. Using eqn (3), to calculate the mean velocity of an ensemble of non interacting particles, it follows that4
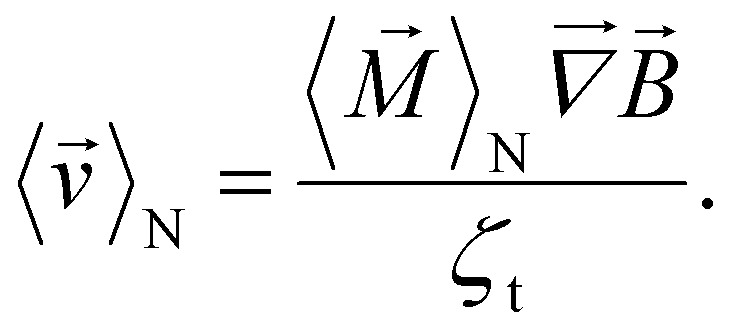


Throughout this work we use the notation 〈*X*〉_N_ for ensemble averaging and 〈*X*〉_T_ for time averaging, where *X* is a random quantity.

As mentioned before, the goal is to improve the separation efficiency of MNPs of different sizes. Thus, we are particularly interested in the velocity ratio and further distance ratio of large (l) to small (s) particles, as a higher ratio indicates a more precise and effective separation. Without a loss of generality, in the following we consider a magnetic field pointing in *x* direction, which also results in a magnetic field gradient along the same axis, leading to5
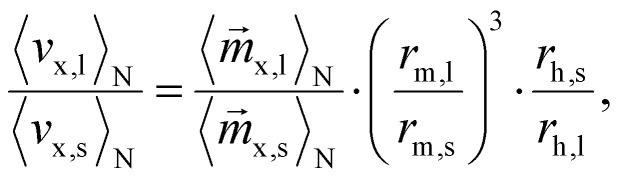
where *r*_h_ and *r*_m_ denote the hydrodynamic and magnetic radii of the particles, respectively. In a commonly used static magnetic field, the normalized ensemble magnetization in external field direction *x*, can be calculated by the well known Langevin function^35^6
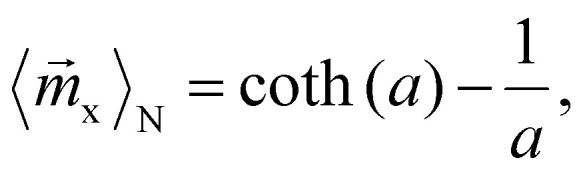
with the Langevin parameter7
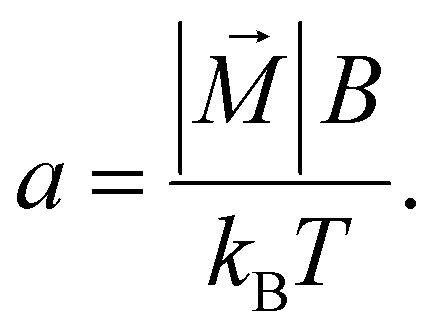


As it can be seen from eqn (5), the velocity ratio depends linearly on the ratio of magnetizations, to the third power on the ratio of the magnetic radii *r*_m_ and linearly on the inverse ratio of the hydrodynamic radii *r*_h_. These size relationships generally result in longer separation times for smaller particles. In other words, larger particles typically move faster than smaller ones^36^ and can therefore reach for example specific target sites sooner. The particle geometry is given by the sample and is therefore fixed, so it is only reasonable to try to modify the first term, the magnetization ratio 
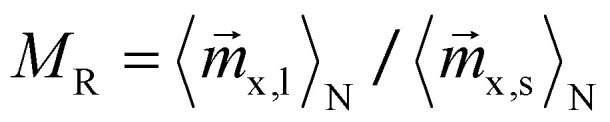
 and boost the size effect even more.

### Basic concept

**Fig. 1:**
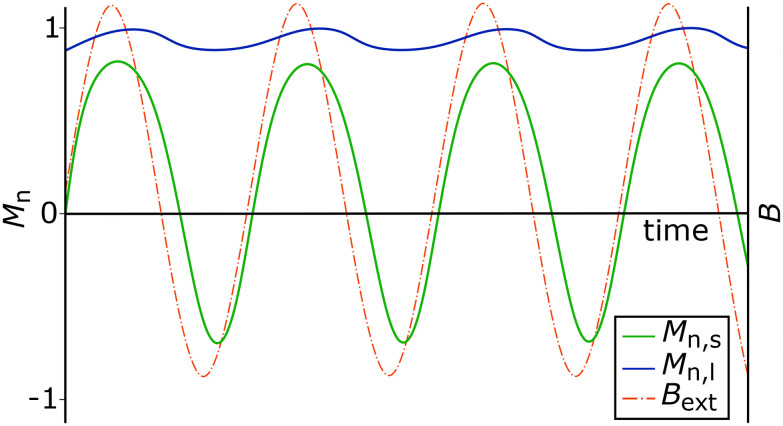
Sketch of the desired magnetization behavior of large (blue) and small (green) particles.

As discussed in the introduction, Coene *et al*.^31^ have already shown that an alternating magnetic field alone can drive MNPs of different sizes into different regimes. In GMS, however, the goal is to guide particles of specific sizes toward designated target locations. This requires a magnetophoretic force, which necessitates a magnetic field gradient. In our dual-field method we try to realize both effects by superimposing a homogeneous alternating magnetic field and an inhomogeneous (gradient) offset field, leading to a total field strength of:

8 *B*_ext_ = *B*_hom,0_sin(2π*ft*) + *B*_grad_,

where *f* is the frequency, *B*_hom,0_ is the amplitude and *t* is the time. The goal is to drive the larger particles toward the target sites, while keeping the smaller ones near their initial positions. Since the magnitude of the magnetophoretic force components depend on the particle's magnetization, the ensemble and time averaged magnetization of the large particles, 
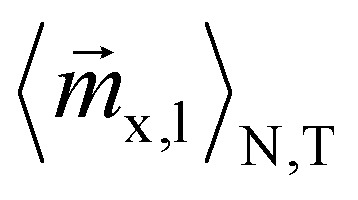
, should significantly exceed that of the smaller ones. Therefore, we aim to drive large and small particles, subjected to *B*_ext_, into two different magnetization regimes and thereby significantly improve the separation efficiency. This desired behavior is illustrated in Fig. 1 and further specified in subsequent sections.

In this context, time averaging refers to averaging over multiple periods of the alternating field, typically on the order of 1 × 10^−4^ s, corresponding to 10 to 100 periods for the frequencies considered in this work. Later in this paper, simulations spanning hundreds of seconds are presented, using these short-time averages as simulation input parameters.

## Model

In this study we consistently use our previously developed and extensively validated Landau–Lifshitz–Gilbert (LLG) model,^37^ also known as egg-model^38,39^ to model the particle dynamics. With this model, the full complexity of internal magnetization dynamics and physical Brownian rotation can be described.

We assume that all MNPs are spherical, single domain particles with uniaxial magnetocrystalline anisotropy. The particles consist of a magnetic core surrounded by a stabilizing non magnetic shell. Each of them has a magnetic moment and can therefore be treated as a small magnetic dipole in a carrier liquid.^40,41^ As the masses of the particles are much larger than those of the solvent molecules, the solvent is considered as thermally equilibrated. Thus, the random collisions between particles and solvent molecules can be modelled as fast fluctuating terms.^42^ We just consider highly diluted MNP suspensions where particle–particle interactions can be neglected.

The orientations of the magnetic moment and easy axis are represented by the direction vectors 
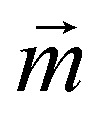
 and 
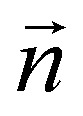
. Internal magnetization dynamics 
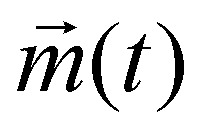
 are modelled by the stochastic LLG equation^43,44^9
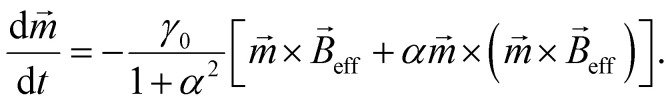


Here *γ*_0_ = 1.7595 × 10^11^ rad T^−1^ s^−1^ is the gyromagnetic ratio and *α* the dimensionless Gilbert damping constant. With *K*_1_ being the anisotropy energy constant, the effective magnetic flux density is given by^44^10
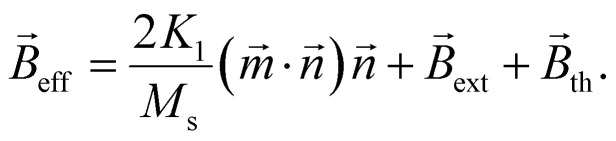

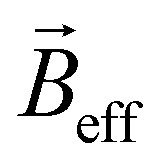
 includes the anisotropy field (first term), the external magnetic field *B*_ext_ and the thermal field *B*_th_. If particles have non vanishing anisotropy energy, the physical rotation of the easy axis and hence of the whole particle is coupled to its internal magnetization 
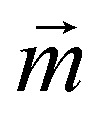
 by the following overdamped differential equation:^44^11
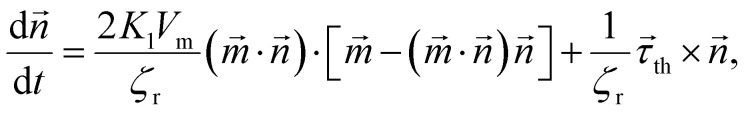
where *τ*_th_ is the thermal torque leading to Brownian rotation, *V*_m_ is the magnetic core volume of the particle and *ζ*_r_ = 8π*r*_h_^3^*μ*_f_ ^45^ the rotational friction coefficient of a sphere in a viscous medium.

All thermal quantities are uncorrelated in space and time and have zero average. Further statistical properties are:^45–47^12
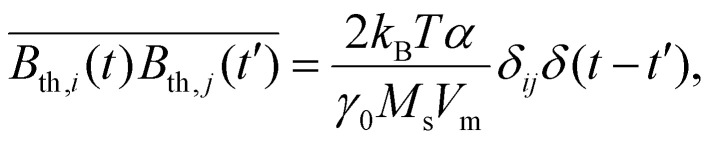
13
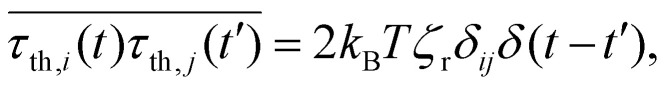
14
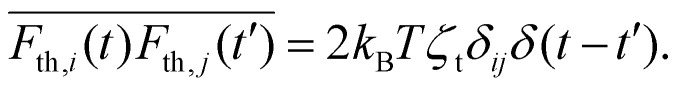
*δ*_*ij*_ is the Kronecker delta, where *i*, *j* correspond to the Cartesian coordinate system *x*, *y*, *z* and *δ*(*t*) is the Dirac delta distribution. The thermal force is implemented by^45^15
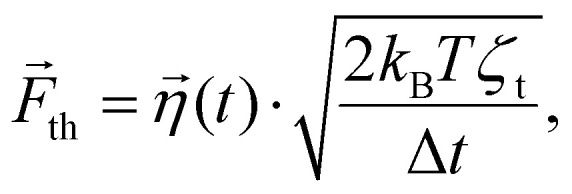
where 
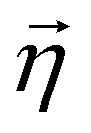
 denotes a random vector with components independently sampled from the standard normal distribution and Δ*t* is the timestep.

Eqn (9) and (11) are integrated numerically with the software presented in ref. 37. In this work we use the adaptive time step Dormand Prince solver with an error tolerance of 1 × 10^−4^, to efficiently perform the time integration of the rotational motion.

Eqn (3) is integrated using a forward Euler method,^48^ with a timestep of 1 s. A comparison between theoretical and simulated diffusion coefficients confirmed that this time step is sufficiently small for our calculations. The theoretical diffusion coefficient was defined as *D* = *k*_B_*T*/*ζ*_t_ and compared with the diffusion coefficient obtained from the time evolution of the particle's mean squared displacement for one spatial direction^45,49^16
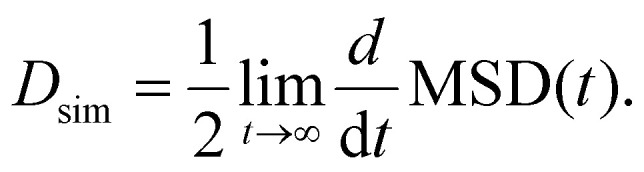


For further details, we kindly refer the reader to our previous work.^37^

## Simulation setup

**Fig. 2:**
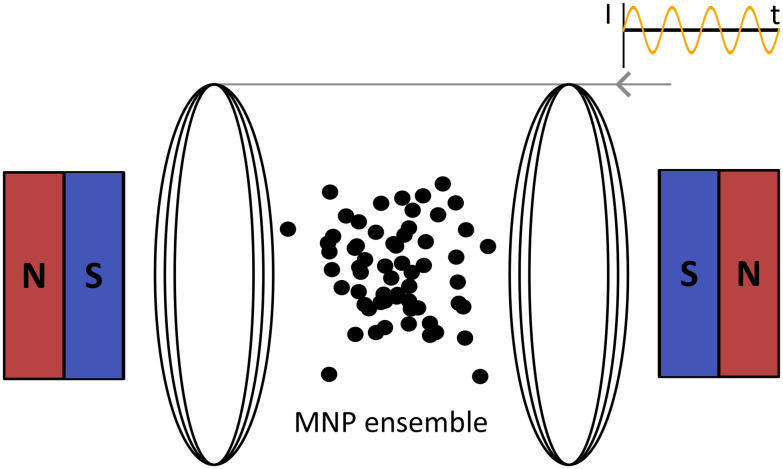
Sketch of experimental setup with an MNP ensemble between AC-driven coils and permanent magnets with opposing south Poles.

The proposed dual-field method requires both a homogeneous magnetic field and a gradient field. A homogeneous alternating field can be generated inside a solenoid or using well known Helmholtz coils. The specific coil parameters are governed by the required magnetic field characteristics, which are determined by the target particle size range for separation. Similarly, a magnetic field gradient can be produced outside a solenoid, by reversing the current in one of the Helmholtz coils (Maxwell coils) or by permanent magnets. However, due to their cost-effectiveness and energy efficiency,^26^ permanent magnets are chosen for the setup, which is illustrated in Fig. 2.

To ensure efficient size separation, the displacement of larger particles due to the magnetophoretic force must significantly exceed the diffusion driven motion of smaller particles. As the subsequent simulations, demonstrating the principal feasibility of our method, consider particles with *r*_m_ = *r*_h_ = 50 nm and 10 nm, the criterion is evaluated accordingly for these sizes. According to the *x* component of eqn (4), with 
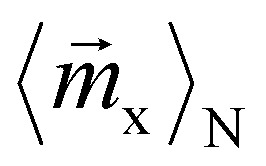
 given by the Langevin function, the mean distance travelled by particles with *r*_m_ = *r*_h_ = 50 nm due to the magnetophoretic force in a magnetic field of 30 mT and magnetic field gradient of 10 T m^−1^ after 1 h is approximately 1 cm. In comparison, the root mean squared displacement (RMSD) due to diffusion in a single spatial direction for particles with *r*_m_ = *r*_h_ = 10 nm is about 0.4 mm. Therefore, we conclude that a magnetic field gradient of about 10 T m^−1^ is sufficient for our simulations as confirmed in the results section. The RMSD was calculated by 
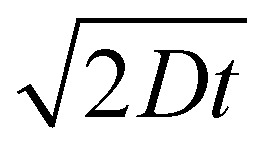
.

To maximize the magnetophoretic force on the particles (eqn (2)), and thereby increase their speed while minimizing separation time, the highest possible magnetic field gradients should be applied. MS techniques typically use inhomogeneous gradients, but these create varying magnetic forces on MNPs, possible leading to inconsistent separation. In contrast, a homogeneous gradient ensures uniform forces, enabling more precise and controlled separation.^29^ However, perfectly homogeneous gradients are difficult to achieve in reality. To demonstrate the feasibility of our method in a realistic setting, we therefore simulate, as introduced earlier, two cylindrical permanent magnets, positioned with identical Poles facing one another.

**Fig. 3:**
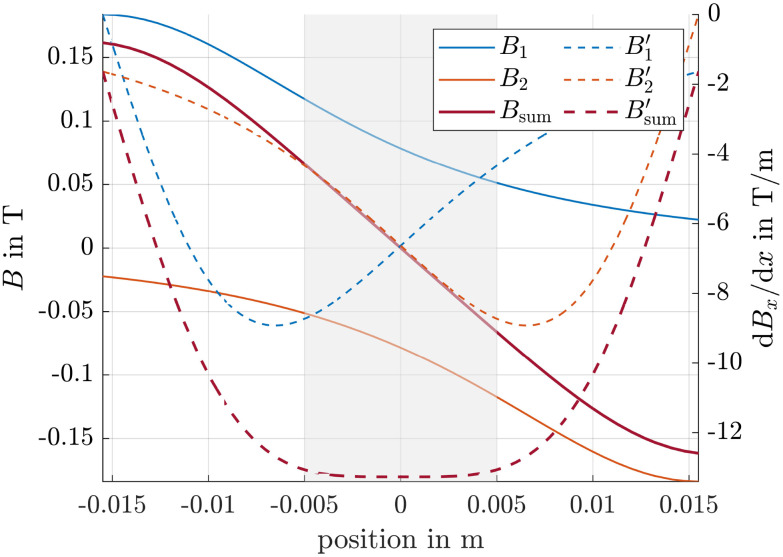
Magnetic flux densities and magnetic field gradients of simulation setup. The subscripts 1 and 2 correspond to the magnets of the setup, the prime in the legend entries represents d*B*_x_/d*x*. The grey shaded area denotes the spatial range considered in subsequent simulations.

The magnetic field along the axis, outside the magnet, can be calculated by^50^17
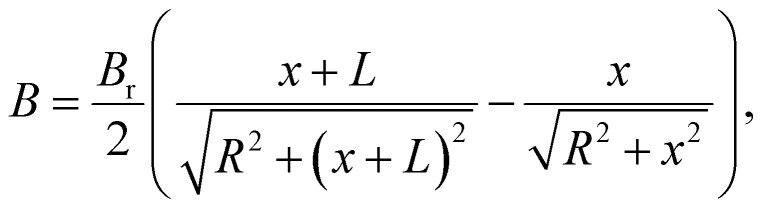
where *B*_r_ is the remanence flux density, *L* is the length and *R* is the radius of the magnet. We chose magnets with *B*_r_ = 1, 3 T, *L* = 5 mm and *R* = 17.5 mm, placed *d* = 31 mm apart from each other, leading to the magnetic fields and magnetic field gradients shown in Fig. 3. The arrangement produces an inhomogeneous field, where the net gradient is significantly larger than the gradient produced by a single magnet alone.^51^ Moreover, the gradient remains nearly homogeneous within the range from −5 mm to 5 mm, with an absolute value exceeding 10 T m^−1^, providing an additional margin of safety with respect to the diffusion criterion. Thus, this region, highlighted in grey, defines the area for following simulations. The absolute value of the magnetic field gradient and thus the speed of the particles is generally increasing with smaller distance between the magnets, but this comes at the cost of reduced homogeneity.

## Results

We begin this section by verifying the basic functionality of our method. We then analyze magnetization ratios for different field strengths and investigate the influence of the alternating field frequency. For demonstration purposes, only two particle sizes with *r*_m_ = *r*_h_ = 10 and 50 nm are considered up to this point. To better reflect the polydispersity of real ferrofluids and demonstrate the general applicability of the dual field method, the size variation is extended to 10, 20, 30, 40 and 50 nm, for the following studies. Next, particle trajectories through the simulation setup are computed, aiming to maximize the travelled distance ratio between large and small particles. We verify that the method remains effective even if *r*_h_≠*r*_m_, as it is the case for realistic core–shell particles. Finally, we demonstrate that our method allows for an arbitrary choice of the separation cut-off radius, confirming that it is not limited to specific particle sizes.

Unless stated otherwise, the following material parameters, similar to those of iron oxide,^31,52^ were used for the simulations: exchange constant *A*_ex_ = 20 pJ m^−1^, *M*_s_ = 4 × 10^5^ A m^−1^, *K*_1_ = 1 × 10^4^ J m^−3^, *α* = 0.1 and *T* = 300 K. Therefore, the particles consist of just one domain^31,53,54^ which matches the model assumptions.

### Proof of concept

To demonstrate that the desired behavior described in section *Basic concept* can principally be achieved, we first consider the simplest case where *r*_m_ = *r*_h_ = 10 and 50 nm, which is in the range of standard particle sizes.^34^ For demonstration purposes, an ensemble of 1000 particles is simulated over 1 × 10^−4^ s, to ensure proper equilibration.

**Fig. 4:**
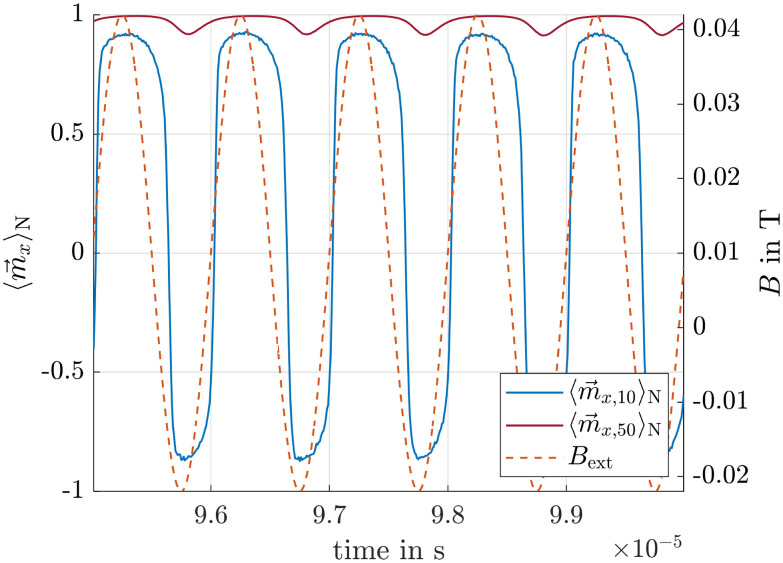
Ensemble averaged vector components of 
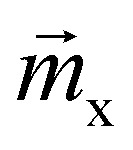
 for particles with *r*_m_ = *r*_h_ = 50 and 10 nm. *B*_ext_ is the resulting magnetic flux density of eqn (8).

As shown in Fig. 4, the ensemble averaged magnetization 
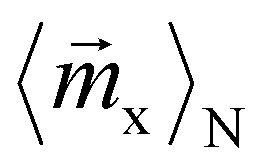
 of 50 nm particles is much higher than that of 10 nm ones. Specifically, the ensemble and time averaged values 
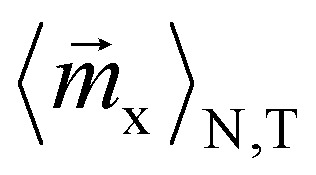
 are 0.97 and 0.23 respectively, resulting in a magnetization ratio *M*_R_ of 4.22. For comparison, the corresponding Langevin magnetizations calculated from eqn (6) are 0.99 and 0.75, leading to *M*_R_ = 1.32. This demonstrates that the magnetization ratio *M*_R_ and consequently the velocity ratio (eqn (5)) can be increased, in this example by a factor of approximately 3.2. We therefore conclude that, in principle, the separation efficiency can be improved by superimposing the gradient field with an alternating homogeneous field, even in magnetic fields strengths where the Langevin function predicts values close to unity.

### Magnetization ratio in various field strengths

**Fig. 5:**
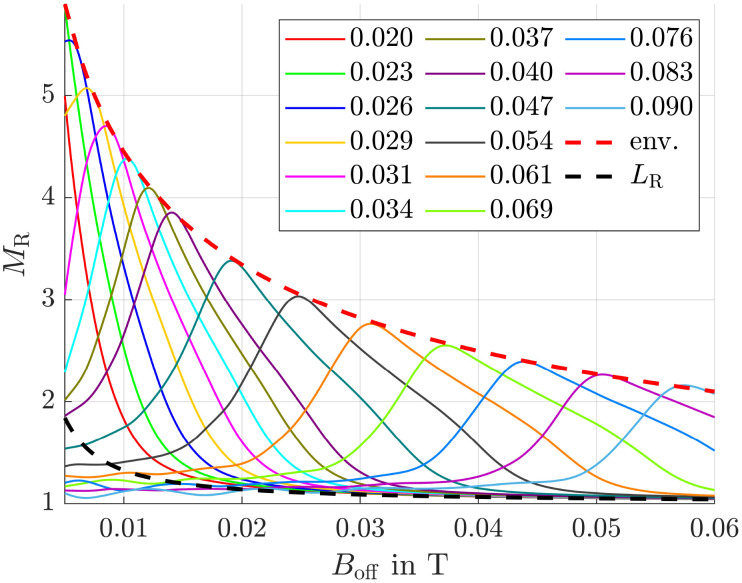
Magnetization ratio for various offset and alternating field strengths with a frequency of 1 MHz. Numbers in the legend represent the amplitude of the alternating field *B*_hom,0_ in T. The red dashed line is the envelope (env.) of the curves.

Since in our simulation setup the offset field strength, predetermined by the permanent magnets, varies relative to the magnets, there exists an optimal combination of offset field strength and adjustable alternating field strength for each position. To determine the maximum magnetization ratio for 50 nm and 10 nm particles at each position, we carried out simulations for various magnetic field combinations. The corresponding results are presented in Fig. 5.

It is clearly visible that for each *B*_off_, there exists an optimal value for the homogeneous field amplitude *B*_hom,0_ and *vice versa*, at which *M*_R_ is maximized. This optimum is indicated by the red dashed envelope in the figure. To demonstrate the efficiency of our method, a black dashed line *L*_R_ is also shown, representing the magnetization ratio calculated using the Langevin function. It is evident that this line lies significantly below the maximum envelope.

**Fig. 6:**
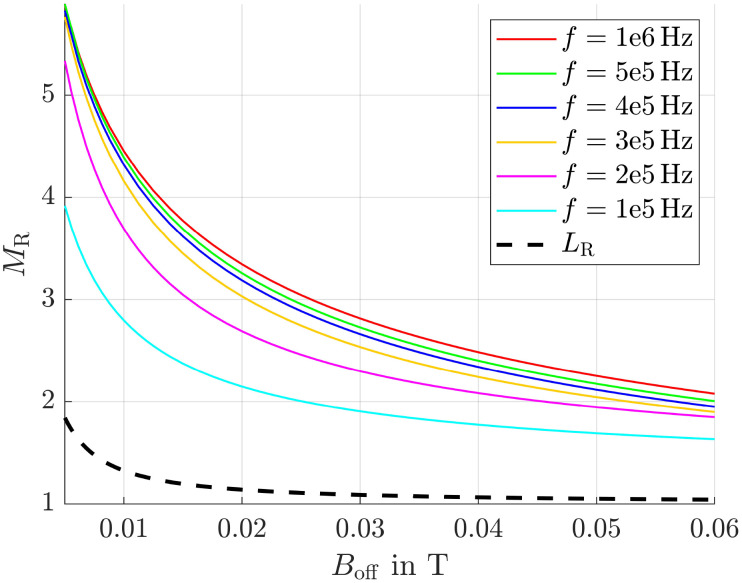
Maximum magnetization ratio (envelopes) for different frequencies. *L*_R_ is the magnetization ratio obtained from the Langevin function in a constant field.

In this paragraph the influence of the alternating field frequency on the magnetization ratio is evaluated. At low frequencies all particles can easily follow the sinusoidal field leading to an ensemble and time averaged magnetization 
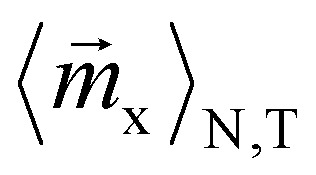
 of approximately zero. In contrast, if the frequency is chosen in a way that small particles can follow the field, whereas large particles can't, 
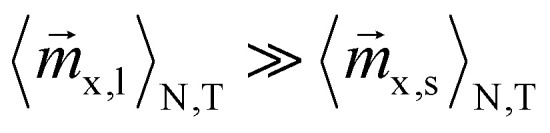
 and the desired effect is reached. In Fig. 6 the envelopes of the magnetization ratios (see Fig. 5) for different frequencies of the alternating field, but the same field strengths are shown. It is evident that *M*_R_ increases with higher frequencies, though the effect tends to saturate at very high frequencies. The most significant improvement occurs between 1 × 10^5^ Hz and 2 × 10^5^ Hz. Additionally, even at the lowest considered frequency, the results significantly outperform those obtained in a constant field (see black dashed line *L*_R_). Since frequencies of *f* = 400 kHz have already been used in previous hyperthermia experiments,^55,56^ this frequency is applied in subsequent simulations.

### Role of anisotropy energy

**Fig. 7:**
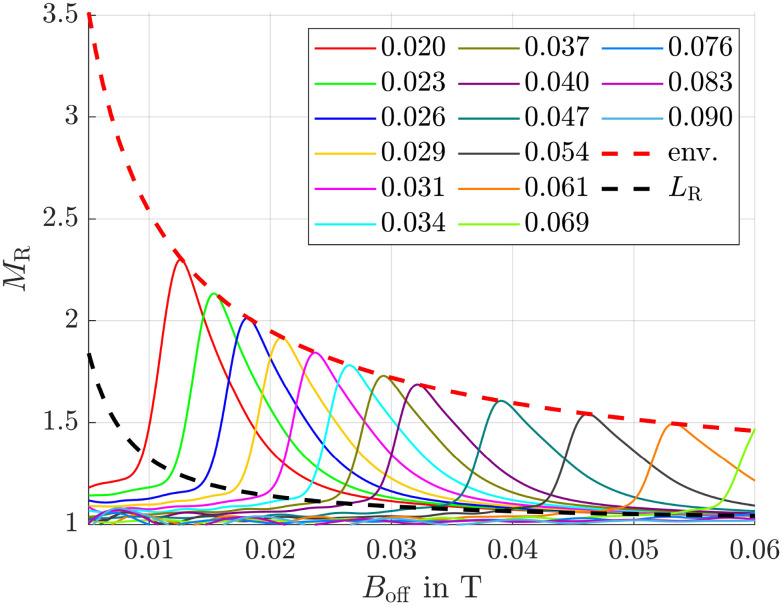
Maximum magnetization ratio for various offset and alternating field strengths with *K*_1_ = 2.5 × 10^3^ J m^−3^ and an alternating field frequency of 1 MHz.

The anisotropy energy constant is a measure of the coupling between a particle's magnetic moment and its crystallographic frame, and therefore strongly influences particle dynamics (see eqn (10) and (11)). This influence becomes evident when comparing Fig. 5 and 7, where the anisotropy constants *K*_1_ were set to 1 × 10^4^ and 2.5 × 10^3^ J m^−3^, respectively. In both cases, the frequency of the alternating magnetic field was 1 MHz. The homogeneous offset field values used in Fig. 7 were chosen to match those in Fig. 5 to facilitate comparison, even though they are clearly suboptimal for the lower anisotropy value.

As a result, the peaks in the magnetization ratio curves shift to higher offset fields. This indicates that for a given *B*_off_, lower alternating field strengths are required to reach the corresponding maximum magnetization ratio when the anisotropy energy is reduced.

**Fig. 8:**
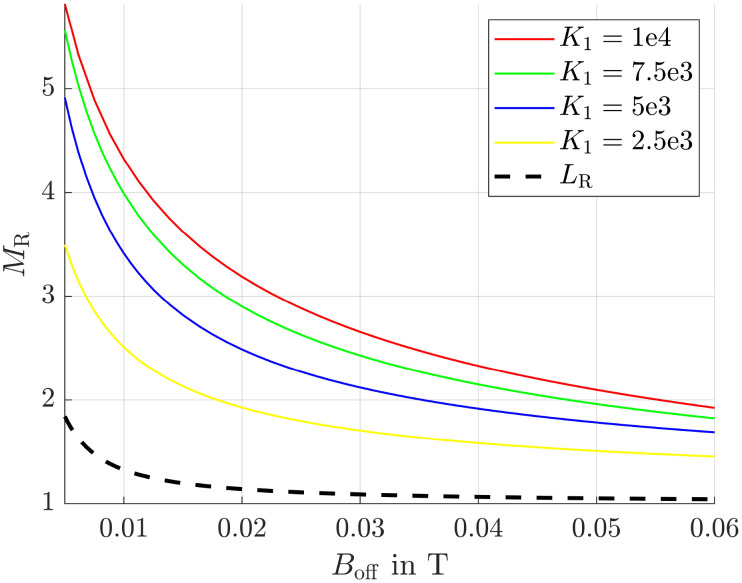
Maximum magnetization ratio (envelopes) for different anisotropy energy constants *K*_1_ in J m^−3^ and an alternating field frequency of 4 × 10^5^ Hz.

Furthermore, Fig. 8, where the field frequency is set to 4 × 10^5^ Hz (as discussed in the previous section), illustrates that the maximum achievable magnetization ratio decreases with decreasing anisotropy energy. This behavior can be attributed to the fact that, at lower values of *K*_1_, the magnetic moments are less tightly constrained to their easy axes and consequently are more free to deviate. As a result, the moments are better able to follow the external magnetic field. Consequently, the characteristic pattern seen in Fig. 4 becomes less pronounced, leading to reduced magnetization ratios.

### Distance evaluation without shell

Up to this point, the primary focus has been on optimizing the magnetization ratio for given offset field strengths, each corresponding to specific positions within the setup. The aim of the next paragraphs is to determine the alternating field strength that maximizes the ratio of the travelled distances between 50 and 10 nm particles at each time step. In other words, the goal is to identify the parameters that enable the most effective size based fractionation.

In Fig. 5 and 6 magnetization ratios are shown, where it is assumed that all particles are at the same position and consequently experience the same offset field. While this assumption is useful for understanding the general behavior and the influence of various parameters, it does not reflect the actual situation during particle motion. As particles of different sizes move at different velocities (see eqn (5)), they gradually separate spatially after leaving the initial configuration. Additionally, Brownian motion contributes further to this separation. To address this, we propose the following scheme to determine the optimal homogeneous field strengths.

The particle positions are computed by integrating eqn (3), with the initial position of all particles set to *x*_0_ = −0.5 mm for all subsequent simulations. The short-time averages, obtained *e.g.* in Fig. 5 and 6 are used directly in the displacement calculations and are assumed to apply instantaneously. This assumption is justified because the timestep in the displacement simulations is 1 s, which is four orders of magnitude larger than the averaging interval in the short-time simulations. The distance simulations have been done with 50 000 particles to ensure robust statistical analysis.

**Fig. 9:**
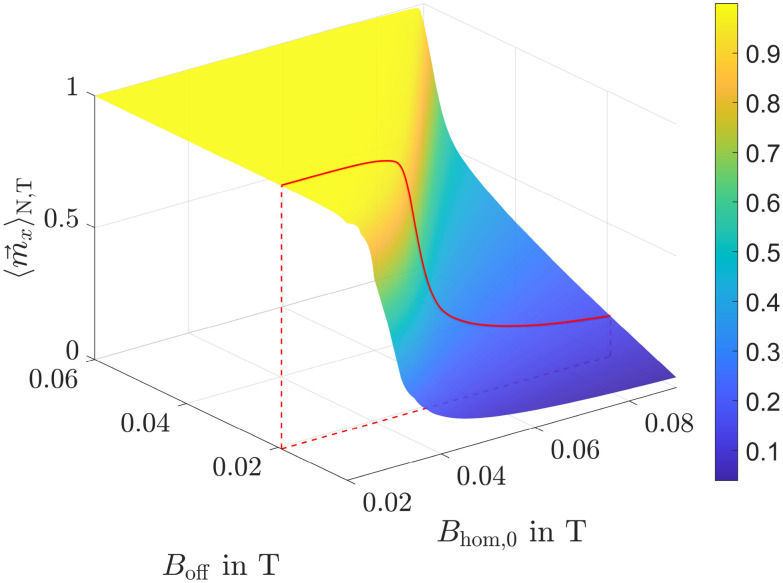
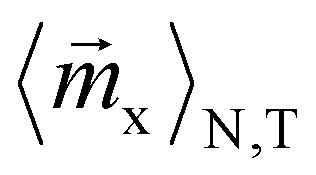
 of particles with *r*_m_ = *r*_h_ = 50 nm as a function of offset and alternating field strength. The solid red line represents the intersection curve after 300 s.

**Fig. 10:**
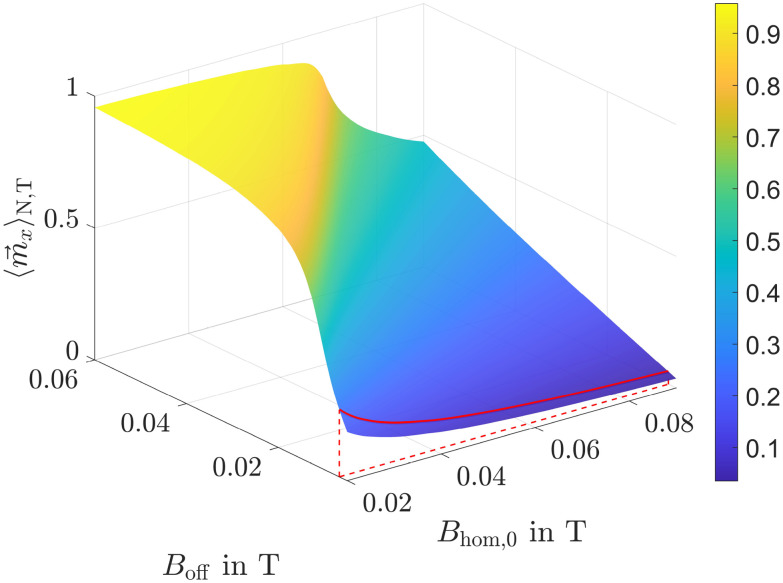
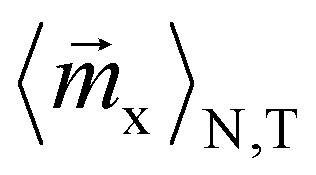
 of particles with *r*_m_ = *r*_h_ = 10 nm as a function of offset and alternating field strength. The solid red line represents the intersection curve after 300 s.

Based on the ensemble and time averaged magnetization results, surfaces are generated through interpolation, illustrating the dependence of 
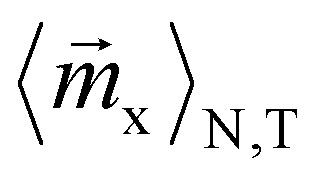
 on both the offset and alternating field strength. Fig. 9 and 10 show such surfaces for 50 and 10 nm particles, respectively.

**Fig. 11:**
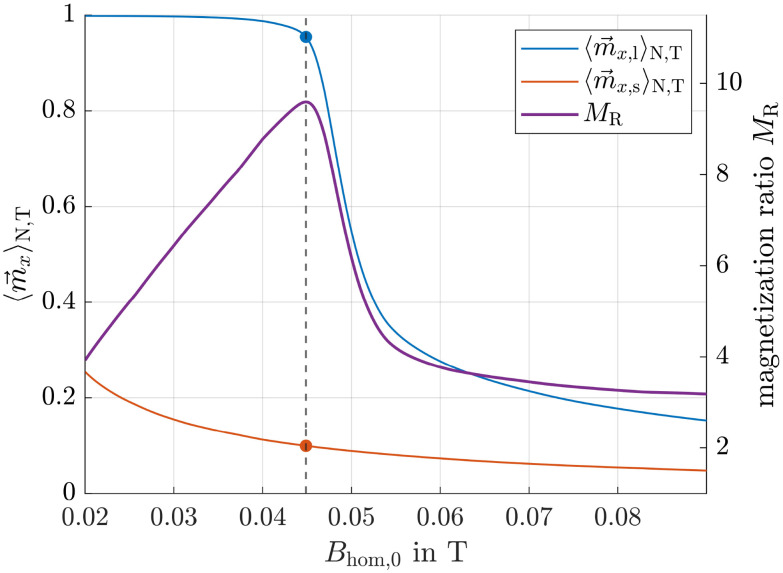
Left axis: surface intersection curves of large and small particles after 300 s. Right axis: magnetization ratio calculated by dividing the intersection curve of large particles by the one of small particles.

The next step is to create intersection curves from the surfaces at the actual mean positions of the particles corresponding to the specific offset field strengths. As an example, the intersection curves for 50 and 10 nm particles after 300 s are explicitly shown in Fig. 11. For a better understanding, these curves are also displayed within the surface plots in Fig. 9 and 10. At this point, the mean position of 50 nm particles is 〈*x*_l_〉_N_ = −1.5 mm, corresponding to an offset field of 20 mT, while the mean position of small particles is 〈*x*_s_〉_N_ = −1.5 mm, corresponding to only 6.6 mT. As also shown in Fig. 11, the maximum magnetization ratio, obtained by dividing the intersection curve of large particles by that of small particles, is achieved at an alternating field strength of approximately 45 mT. This value represents the optimal field strength for the current particle positions and current simulated time.

**Fig. 12:**
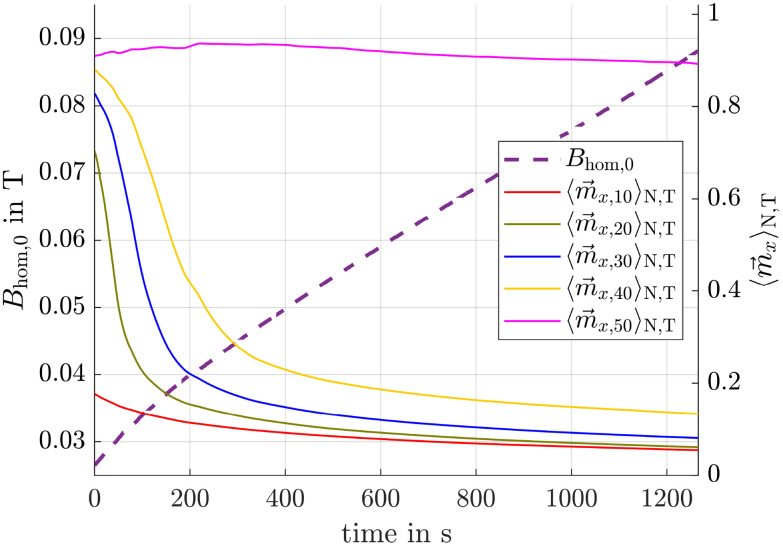
Left: Time evolution of the alternating magnetic field strength (dashed line). Right: 
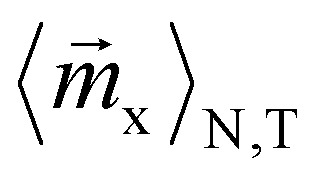
 over time for different particle radii, resulting from the time varying alternating magnetic field superimposed with the offset field (solid lines).

To reflect a more practical application scenario, we show how adapting the alternating field strength over time, based on the evolving mean particle positions, can enhance separation performance. For this purpose, the procedure described above is repeated at every timestep. The resulting time course of the alternating field strength, shown in Fig. 12, for a total simulated time of 1269 s, illustrates that a dynamic adjustment can significantly improve the magnetization ratio between larger and smaller particles. Because larger particles move more quickly toward regions with higher offset fields, the alternating field strength can be increased without significantly reducing 
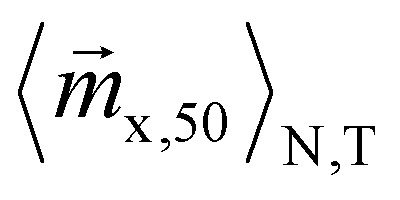
. Since smaller particles remain longer in regions with lower offset field strengths, the increasing alternating field gradually dominates over the offset field as the simulation progresses, driving 
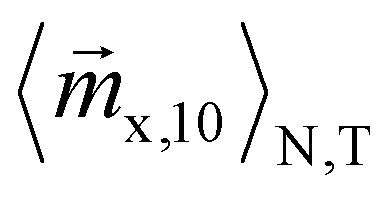
 towards zero. This dynamic further enhances the magnetization ratio, yielding even higher values than those observed in Fig. 5.

The time varying field strength leads to the ensemble and time averaged magnetizations shown in Fig. 12. It can be observed, that 
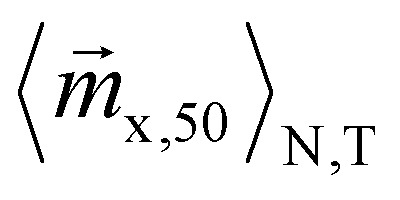
, resulting from the dual field method, stays nearly aligned with the field direction, whereas 
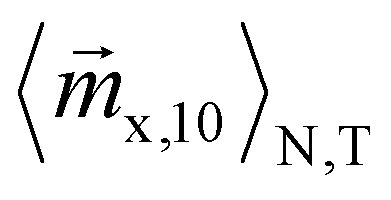
 remains close to zero. This is exactly the intended outcome, as it indicates that larger particles will consistently move toward the magnets with minimal influence from the alternating field, while smaller particles will remain almost stationary at their initial position.

So far we have demonstrated our method using particles with radii of 10 nm and 50 nm. However, since real ferrofluids are polydisperse, in the following sections we will also explore how the method performs for particles with *r*_m_ = *r*_h_ = 20, 30 and 40 nm. Although real particle size distributions are continuous, a discrete set of sizes was selected for illustrative purposes.

Even though the optimization of the alternating field strength was performed using particles with *r*_m_ = *r*_h_ = 10 nm and 50 nm, Fig. 12 clearly shows that the time averaged magnetization of other particle sizes is also significantly reduced compared to the 50 nm particles and to their corresponding Langevin magnetization. In this simulation, the Langevin magnetization resulting solely from the gradient field lies between 0.7 and 0.8 for 10 nm particles, while for the other particle sizes it ranges from 0.95 to 1. To not overload the figure, these reference values are not explicitly shown.

**Fig. 13:**
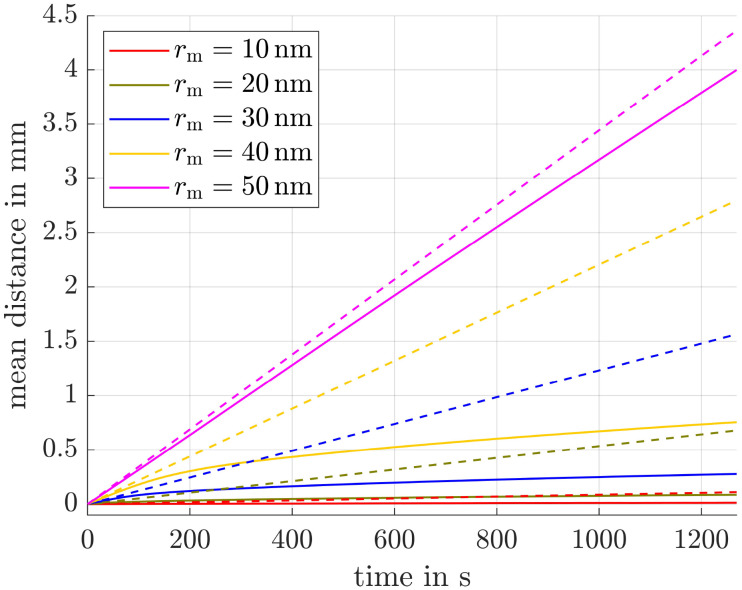
Mean travelled distances of particles of different size resulting from our method (solid lines) and a gradient field only (dashed lines).

At this point we introduce the separation cut-off radius *r*_cut_, which defines that particles with radii equal to or greater than *r*_cut_ should be separated from smaller ones. As the text above indicates, *r*_cut_ = 50 nm is used in this and in the following sections unless specified otherwise. Next, the resulting mean absolute travelled distances of particles with different radii, obtained using the dual field method, are compared to those obtained using a gradient field only. The results are shown in Fig. 13. As can be seen, for particles with *r* = 50 nm, the travelled distances are at the same scale. Under a gradient field alone, they travel 4.36 mm, while in the presence of the combined fields, the travelled distance slightly decreases to 4.00 mm, leading to a distance ratio of 1.09. This minor reduction is expected, as Fig. 12 and the accompanying explanation indicate that the magnetizations remain at similar magnitude in both cases. However, for smaller particles with *r* = 10 nm, the difference is much more pronounced. When subjected only to a gradient field, they travel 0.11 mm, whereas under the combined fields, their travelled distance is drastically reduced to just 0.013 mm, corresponding to a reduction by a factor of 8.34. The ratio of travelled distances between 10 nm and 50 nm particles in a gradient field is approximately 39, whereas in the combined fields, this ratio increases to 302, an enhancement by a factor of approximately 7.7.

As previously mentioned, the evolution of the alternating magnetic field strength was optimized using the magnetization ratio of 10 nm and 50 nm particles. Nevertheless, the separation of 50 nm particles from other sizes has also been significantly improved. Specifically, 40 nm particles travelled 0.76 mm instead of 2.80 mm under a gradient field alone, resulting in a distance ratio of 3.70. Similarly, 30 nm particles travelled 0.28 mm instead of 1.57 mm, yielding a distance ratio of 5.63 while 20 nm particles travelled only 0.087 mm compared to 0.68 mm corresponding to a distance ratio of 7.84. All values are summarized in Table 1.

**Table 1:** Mean travelled distances of particles with different radii after 1269 s in mm, comparing the results from using a gradient field only and the dual method (*r*_cut_ = 50 nm)

*r*_m_ (nm)	Gradient field (mm)	Combined fields (mm)	Distance ratio
50	4.36	4.00	1.09
40	2.80	0.76	3.70
30	1.57	0.28	5.63
20	0.68	0.087	7.84
10	0.11	0.013	8.34

This demonstrates that the additional alternating field significantly improves separation efficiency by slowing down particles smaller than 50 nm while allowing 50 nm particles to remain nearly unaffected, ultimately enhancing the separation process.

### Distance evaluation with shell

For simplicity, we have so far focused on particles without a shell, assuming *r*_h_ = *r*_m_, while being aware that this would lead to colloidal instability in real suspensions. In practical ferrofluids, magnetic cores are coated with stabilizing surfactant layers, leading to *r*_h_ = *r*_m_ + *s*, where *s* is the shell thickness. In this section, we demonstrate that our method remains effective even under these more realistic conditions.

**Fig. 14:**
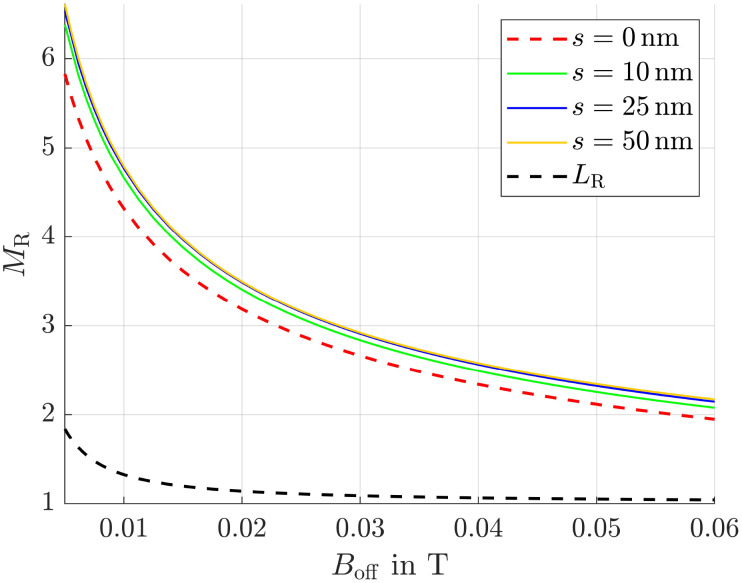
Maximum magnetization ratio (envelopes) for different shell thicknesses. The red dashed line is the one from Fig. 5 for shell thickness *s* = 0 nm, *L*_R_ is the magnetization ratio obtained from the Langevin function in a constant field.

Therefore, we did the same simulations as before, this time using shell thicknesses *s* of 10, 25, and 50 nm, and generated the envelopes of the magnetization ratio as in Fig. 5. The results are shown in Fig. 14, where it can be seen, that all curves corresponding to *s* > 0 nm lie above the red dashed line, which represents *s* = 0 nm. This suggests an even higher distance ratio between 50 and 10 nm particles compared to the non-shell case.

Next, we recalculated the travelled distances, this time assuming a uniform shell thickness of *s* = 25 nm for all particle sizes. As before, the optimized time course of the alternating field strength was determined to maximize the distance ratio between particles with *r*_m_ = 10 nm and *r*_m_ = 50 nm.

To enable a meaningful comparison with the previous results, the mean travelled distances were evaluated at the moment when the 50 nm particles under the combined fields, reached a distance of 4 mm. This corresponds to simulated times of 1269 s for the uncoated particles and 1861 s for the coated ones. Due to the increased hydrodynamic radii from the shells, the translational friction coefficients rise, leading to slower particle motion. Consequently, it is necessary to extend the simulated time in the case of coatings to 1861 s.

As anticipated from Fig. 14, the distance ratio between 10 nm and 50 nm particles increased further compared to the uncoated case. Specifically, the ratio in a gradient field only is approximately 92 and under dual fields 745, an enhancement by a factor of approximately 8.1. This demonstrates that our method remains effective and applicable even under these more realistic conditions. Since the distance plot for coated particles closely resembles the one without coatings shown in Fig. 13, it is omitted here. Instead, the mean travelled distances are summarized in Table 2.

**Table 2:** Mean travelled distances of particles with different radii and *s* = 25 nm after 1861 s in mm, comparing the results from using a gradient field only and the dual method (*r*_cut_ = 50 nm)

*r*_m_ (nm)	Gradient field (mm)	Combined fields (mm)	Distance ratio
50	4.27	4.00	1.06
40	2.52	0.70	3.59
30	1.25	0.24	5.20
20	0.44	0.062	7.14
10	0.046	0.0052	8.90

**Fig. 15:**
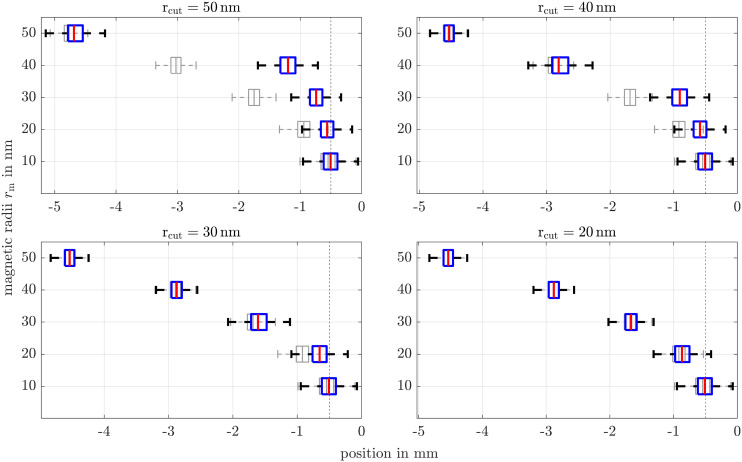
Box plots of particle end-positions in the simulation setup. The blue boxplots correspond to the combined fields method, whereas gray boxplots correspond to a gradient field only. Whiskers are limited to 1.5 times the interquartile range. To not overload the figure outliers are not shown explicitly. The dashed lines indicate the initial positions.

Finally, we demonstrate that the dual field method is not limited to separating 50 nm particles from smaller sizes (*r*_cut_ = 50 nm), but that the separation cut-off radius can be chosen arbitrarily. The magnetic field profile is tailored accordingly to maximize the magnetization ratio defined as 
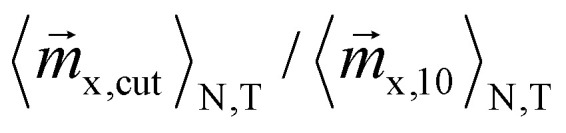
. Fig. 15 presents the final positions of coated particle ensembles of various sizes, after 1861 s for different values of *r*_cut_. The results compare particles subjected to both, dual fields (blue) and gradient field alone (gray). It is evident that particles with *r*_m_ ≥ *r*_cut_ are significantly better separated from smaller ones using the dual field method, compared to the conventional GMS approach. For the case where *r*_cut_ = 20 nm the separation effect is not visible at the spatial and temporal scales shown in the figure. However, the dual field method remains effective in principle for this size range as well. It is also important to note that the optimal alternating magnetic field strength needed for separation is strongly dependent on the chosen cut-off radius. For example, while a field strength of up to 88 mT is necessary for optimal separation if *r*_cut_ = 50 nm (Fig. 12), only 33 mT are sufficient when *r*_cut_ = 20 nm.

## Discussion

Inspired by prior concepts of size-dependent magnetization dynamics, this work presents a method to enhance conventional GMS methods through the superposition of an alternating homogeneous and gradient magnetic field. The aim was to improve the size fractionation of MNPs by exploiting the difference in magnetization dynamics of particles of varying sizes.

After a verification of the basic concept, simulation results showed that the magnetization ratio between 50 and 10 nm particles strongly depends on the combination of offset and alternating magnetic field strengths. For each offset field an optimal alternating field strength exists where *M*_R_ is maximized, clearly exceeding the values resulting from the Langevin function. Additionally, frequency and anisotropy energy play a crucial role: higher frequencies lead to larger magnetization ratios with the most pronounced improvement occurring between 100 and 200 kHz. Although, largest *M*_R_ are reached at 1 MHz, to stay as realistic as possible, 400 kHz have been chosen for subsequent simulations. Lower anisotropy energy weakens the alignment between magnetic moments and their easy axes. As a result, the peaks in magnetization ratio shift to higher offset field values, indicating that smaller alternating field amplitudes are sufficient to reach the corresponding maximum *M*_R_ at a given *B*_off_. Overall, the maximum achievable *M*_R_ tends to decline as the anisotropy energy decreases.

In the following distance simulations, 50 nm particles moved effectively through the setup, showing minimal influence from the additional alternating magnetic field. In contrast, smaller particles exhibited a substantial reduction in mobility when exposed to the combined fields, leading to a significantly increased distance ratio. Notably, this effect was not limited to the smallest and largest particles but extended across a broad range of intermediate sizes. By continuously adjusting the alternating magnetic field strength based on mean particle positions, the method dynamically adapts to evolving system conditions. This method effectively slows down smaller particles even more while enabling larger ones to advance, resulting in an efficiency and resolution improvement of size-based separation compared to the use of a gradient field alone.

To test the method under more realistic conditions, we repeated the simulations using non-zero shell thicknesses of 10, 25 and 50 nm to account for stabilizing coatings commonly present in practical ferrofluids. The resulting magnetization ratios were consistently higher than in the uncoated case, indicating further improved separation. Using a uniform shell thickness of *s* = 25 nm for all particle sizes, we re-evaluated the mean travelled distances and observed, as expected, an increased distance ratio between 10 nm and 50 nm particles.

Finally, we proved that the dual field method is not limited to specific particle sizes, but we showed that the separation cut-off radius can be chosen arbitrarily according to any particle size distribution. The particle end-positions, visualized in the box plots, show clearly that particles where *r*_m_ ≥ *r*_cut_ has been well separated from other sizes, demonstrating the predominance of our method compared to the conventional GMS method. The results also confirm that the dual field method remains highly effective under more realistic conditions and is well suited for practical applications involving coated nanoparticles and particle size distributions.

At the beginning of the section *Simulation Setup*, we showed that applying a magnetic field gradient of 10 T m^−1^ results in a magnetophoretic displacement of 50 nm particles that is 25 times larger than the RMSD of 10 nm particles due to diffusion. This indicates that diffusion can be effectively overcome by magnetophoretic motion. Achieving such a large displacement ratio, however, requires relatively high magnetic field gradients, which in turn necessitate strong magnetic fields. As shown in Fig. 5, achieving high magnetization ratios in such strong gradient fields also demands strong homogeneous alternating fields. Reducing this ratio could lower the required gradient and consequently the necessary field strengths, making the method easier to apply in experiments. The trade-offs would be slower particle motion leading to longer separation times and greater influence of diffusion. Nevertheless, this method merits further investigation.

While this study focuses on non-interacting particles, dipole–dipole interactions, especially at higher volume concentrations, can significantly affect particle and magnetization dynamics and thus magnetization ratios. These effects are expected to adversely impact separation efficiency in general. However, further investigations are required to determine their specific influence on conventional GMS compared with the proposed dual-field method.

Although this work provides a strong theoretical and computational basis, future experimental validation is essential. Moreover, optimizing the spatial configuration of magnets or exploring time varying gradient profiles could further boost performance. Overall, the presented dual field method offers a significant advancement in GMS technology, which could lead to narrower particle size distributions. An important step towards improving the precision of biomedical applications such as targeted drug delivery, imaging and hyperthermia.

## Conclusion

This study introduces a novel dual magnetic field method to enhance GMS by superimposing a homogeneous alternating magnetic field onto an inhomogeneous offset field. The method significantly increases the magnetization ratio and following the velocity- and distance ratio between MNPs of different size. The technique remains effective even for more realistic coated particles and for various separation cut-off radii, demonstrating its robustness and practical relevance. Overall, the study contributes a fundamentally new and scalable strategy to achieve high resolution MNP size fractionation, addressing a critical bottleneck in both biomedical and technical applications.

## Author contributions

Manuel Wolfschwenger: Conceptualization, methodology, software, writing – original draft. Jonathan Leliaert: Conceptualization, writing – review & editing Aaron Jaufenthaler: Conceptualization, writing – review & editing. Daniel Baumgarten: Conceptualization, writing – review & editing, supervision.

## Conflicts of interest

The authors declare no conflict of interest.

## Data Availability

The code for the simulation model can be found at https://github.com/ManuelWolfschwenger/MD_Sim_Wolfschwenger this link GitHub Repository with https://doi.org/10.5281/zenodo.15696940.
